# How Digital Storytelling Applied in Health Profession Education: A Systematized Review

**DOI:** 10.30476/jamp.2021.87856.1326

**Published:** 2021-04

**Authors:** RITA MOJTAHEDZADEH, AEEN MOHAMMADI, AMIR HOSSEIN EMAMI, AFAGH ZAREI

**Affiliations:** 1 Department of E-learning in Medical Education, Virtual School, Tehran University of Medical Sciences, Tehran, Iran; 2 Department of Hematology/Oncology, Imam Khomeini Hospital, Tehran University of Medical Sciences, Tehran, Iran; 3 Department of Medical Education, Medical School, Tehran University of Medical Sciences, Tehran, Iran

**Keywords:** Health education, Medical education, Nursing education

## Abstract

**Introduction:**

Storytelling is one of the earliest ways to share scientific advancements and discoveries. The advent of technology has updated this ancient art into a digitalized form. The boundaries between the digital storytelling (DST), and other types of videos are unclear. Therefore, in this review, the process, aim, producers, and uses of DST in health profession education have been reviewed.

**Methods:**

This study is a systematized review, which is in nature like a systematic review with only a few differences in the comprehensive search and quality assessment procedure. All studies, whose duplicates were removed, were retrieved from Science Direct, PubMed, and Scopus databases or through google scholar search engine screened in 3 stages: title, abstract and full study. All journal articles including experimental, case study and case report, mixed method, and qualitative studies in English language in the field of health profession education were chosen for this review after being evaluated based on QUESTS dimensions.

**Results:**

In total, 35 articles were included in the review. The studies had been done in health promotion, nursing education, medical education, patient education, social work education, and community health education. In some of these studies, the producers and users of digital stories were different, which is in contrast with center for digital storytelling that emphasizes the process of DST. The results of this review showed that all stakeholders of health system could be producers of digital stories with various aims; e.g. community health, empathy promotion, attitude and behavior change, clinical thinking, and skills improvement.

**Conclusion:**

This systematized review indicated that DST has some applications in different subjects in different fields of health professions and with a potential to be used by different stakeholders of health system. According to the definition of DST, digital storytelling involves the process of writing a script to produce a digital story by one individual or a group. Consequently, there is a difference between DST and producing a digital story. Therefore, researchers should consider the correct use of this term in their studies. Although few interventional and high-quality studies have been conducted in this area, further quantitative and qualitative research is suggested.

## Introduction

Digital storytelling (DST) is a combination of the storytelling and digital components including texts, pictures, recorded audio narrations, music and videos ( 1 ). As the story of Sinuhe in ancient Egypt, the story of Homer in Greece, or the legends of the early humans suggest, no one can deny the role of storytelling as the basis of human communication, for this oral tradition has contributed to the teaching and transferring knowledge, skills, attitudes, and values ( 2 , 3 ). Storytelling and learning are inextricably intertwined because the process of writing a story is a process of producing meaning, and accordingly, it is a new notion in the field of education ( 4 , 5 ). In health practice which students have daily encounters to plenty of client’s stories ( 6 ), storytelling could be a potential tool to obtain the educational goals especially critical thinking ( 7 ), the central goal in any educational system ( 8 ).

However, with the advent of technology, this ancient art has gained a digitized mode ( 2 ). In the late 1980s, DST movement emerged in the Center for Digital Storytelling (CDS), a non-profit community art organization in Berkeley, California by Joe Lambert that had an experience in theater. CDS developed components of DST, which are self-revelatory, personal or first person voice, live experiences, photos more than moving images, soundtracks, length and design, and intention. According to CDS, these elements define DST ( 9 ).

Lambert also mentioned seven steps in digital storytelling in story circle. These steps are owning your insights, finding the moment, seeing, hearing, assembling and sharing your story ( 9 , 10 ). Although CDS has raised seven steps for digital storytelling, Robin (2016) considered DST in a 12-step process for educators including choosing a topic, conducting research on the topic, writing the first draft of the script, receiving feedback on the script, revising the script, finding, creating and adding images, respecting copyrights, creating a storyboard, recording audio narration, adding background music (optional), building the digital story, and publishing it. For educational uses, Robin has considered a three- to eight-minute length ( 1 ).

In addition to what Lambert ( 9 ) and Robin ( 1 ) have done, the literature shows various designs of DST. For example, Schuck and Kearney ( 11 ) presented the steps of capturing pedagogical frame and developing the idea, structuring story board, arranging storyboard, preparing the video, video-recording, arranging the video, presentation of the video to a small group, presentation of the video to general audience/classroom, and dissemination. 

Nowadays, as we explore the “digital storytelling” topic on the Internet, we face a volume of studies as well as books that have introduced this technology in various subject instructions. It means DST has become a powerful educational technology for teaching and learning in recent years ( 12 ) in which higher order thinking (HOTS) skills of Bloom digital taxonomy ( 13 ) are used. According to these studies, by making a digital story, students involve in reflection ( 14 - 16 ), sharing ideas and forming learning communities ( 12 , 17 ), increased levels of active participation ( 16 , 18 ), meaning making and making progress in multiliteracy like digital literacy, global literacy, technology literacy, visual literacy, and information literacy ( 10 ). Since one of the stages of DST involves sharing digital stories, there is an opportunity for all learners to share their experiences and receive peer feedback ( 14 , 19 , 20 ). Hence, collaborative learning and social learning are facilitated through DST ( 12 ). Due to the development of question making skills, organizing ideas, expressing opinions, and constructing meaningful narratives ( 12 ), the skill of interpersonal communication ( 21 , 22 ) and empathy ( 21 , 23 ) are promoted. 

All advantages mentioned above for DST confirm the personal nature and first-person voice of digital stories that CDS regards as essential elements of digital storytelling ( 9 ) and a critical issue in learning process. In spite of this, in educational uses of DST, Robin (2006) refers to decision making and those responsible for DST, an instructor or a student ( 10 ). When the instructor uses DST, the students only see the product without engagement in the DST process. Consequently, first person narrative is produced by the teacher not the students, so it is possibly hard to achieve many of the benefits of digital storytelling. On the other hand, there are some evidence that the boundary between DST and any other videos which are shared in online environments is clear-cut ( 1 , 14 , 24 , 25 ). There are some systematic reviews conducted on DST; for instance, de Jager, et al. (2017), who conducted a systematic review of digital storytelling in research ( 26 ), Stargatt et.al (2019), who introduced their review protocol to investigate the health-related outcomes of DST on elderlies engaged in digital storytelling ( 27 ), and Moreau (2018), who examined the contexts and goals of implementation of DST in health profession education ( 28 ). However, it is still unknown how and by whom the digital storytelling has been used in health education. 

In this perspective, the aims of this review in health education include identifying: 1- what steps of DST are used? (process) 2- who do the digital storytelling? (producers, learner or others) 3- what are the educational implications of digital storytelling and 4- what fields of health profession have used DST? (field of study)

## Methods

This study is a systematized review like a systematic review except that some of its components are omitted. According to Grant (2009), a systematized review attempts to include one or more elements of a systematic review when researchers do not have access to all resources required for a systematic review. The researcher may or may not include a comprehensive search or quality assessment ( 29 ). 

To find literature about the implications and the methods of DST in health education, the e-search was performed using PubMed, Science Direct and Scopus. The search was also performed with Google Scholar search engine. 

The following search terms “Digital Storytelling” and “Health Profession Education” were used based on the research questions in English-language literature with no time limitation in February 2020. We also used advanced search options and Boolean operators ‘AND’ and ‘OR’ and search strategies. For illustration, PubMed was searched with this strategy: (("Health Education"[Mesh] OR "Health Education, Dental"[Mesh] OR "Education, Public Health Professional"[Mesh]) OR ( "Education, Medical"[Mesh] OR "Education, Medical, Undergraduate"[Mesh] OR "Education, Medical, Graduate"[Mesh] OR "Education, Medical, Continuing"[Mesh] )) OR ( "Education, Nursing"[Mesh] OR "Nursing Education Research"[Mesh] OR "Education, Nursing, Continuing"[Mesh] OR "Education, Nursing, Graduate"[Mesh] OR "Education, Nursing, Baccalaureate"[Mesh] OR "Education, Nursing, Associate"[Mesh] OR "Education, Nursing, Diploma Programs"[Mesh] ) AND Digital storytelling. 

The study selection was done for data collection and all records were checked for duplications using Endnote. The EndNote software (version X9) was used to manage the included as well as excluded articles in the research process. The following criteria were considered for the inclusion of papers in this review:

- Paper format: journal article
- Type of article: experimental, case study and case report, mixed method and qualitative study
- Paper language: English
- Paper subject: Digital Storytelling in Health Profession Education

After that, the screening was done at three levels: title, abstract, and full study. At level one, irrelevant papers to health sciences were excluded. At level two, abstracts were reviewed according to exclusion criteria to choose relevant papers. The exclusion criteria were the review articles, books, abstract only, comments or letters, and languages other than English. Finally, the full texts were assessed based on QUESTS dimensions (Table 1 and 2), and the eligible articles were found. In addition, some studies were found through hand searching, which were evaluated, too. 

**Table 1:** The QUESTS Dimensions for Evaluating Evidence in Educational Practice

Quality	How good is the evidence?
Utility	To what extent can the method be transferred and adopted without modification?
Extent	What is the extent of the evidence?
Strength	How strong is the evidence?
Target	What is the target? What is being measured? How valid is the evidence?
Setting	How close does the context or setting approximate? How relevant is the evidence?

**Table 2:** The Evaluation of Retrieved Papers in Term of QUESTS Dimensions

Name of study	Author/ year	Quality	Utility	Extent	Strength	Target	Setting
Bridging storytelling traditions with digital technology	Cueva, M./2013 (31)	B	C	A	B	C	C
A CBPR^1^ approach to finding community strengths and challenges to prevent youth suicide and substance abuse	Holliday, C.E./ 2016 (32)	B	C	A	A	B	B
Design, Implementation, and Lessons Learned from a Digital Storytelling Project in an Undergraduate Health Promotion Theory Course	Rimando, M./ 2015 (33)	B	C	A	A	B	C
The power of digital storytelling as a culturally relevant health promotion tool	Briant, K.J./ 2016 (34)	B	C	A	A	B	B
Digital storytelling: a tool for health promotion and cancer awareness in rural Alaskan communities	Cueva, M./ 2015 (35)	C	C	A	A	B	B
Digital storytelling as a narrative health promotion process: Evaluation of a pilot study	DiFulvio, G.T. / 2016 (36)	C	C	A	B	A	C
Puerto Rican Latina youth coming out to talk about sexuality and identity	Fiddian-Green, A./ 2017 (37)	B	C	A	A	B	A
From intervention to invitation: reshaping adolescent sexual health through storytelling and games	Gilliam, M./ 2012 (38)	B	C	B	A	C	A
Stories for change: Development of a diabetes digital storytelling intervention for refugees and immigrants to Minnesota using qualitative methods Health behavior, health promotion and society	Njeru, J.W./ 2015 (39)	B	C	A	B	B	B
Imagine HEALTH: Results from a randomized pilot lifestyle intervention for obese Latino adolescents using Interactive Guided Imagery	Weigensberg, M.J./ 2014 (40)	B	B	A	C	C	A
Promoting Positive Youth Development and Highlighting Reasons for Living in Northwest Alaska Through Digital Storytelling	Wexler, L./ 2013 (41)	B	C	A	B	B	B
Pilot feasibility study of a digital storytelling intervention for immigrant and refugee adults with diabetes	Wieland, M.L./ 2017 (42)	C	C	A	B	A	B
Efficacy of rational emotive digital storytelling intervention on knowledge and risk perception of HIV/AIDS^2^ among schoolchildren in Nigeria	Ezegbe, B./ 2018 (43)	C	C	A	C	B	B
Digital storytelling: An emergent method for health promotion research and practice	Gubrium, A.C./ 2019 (44)	B	C	A	A	A	C
People of immigrant and refugee background sharing experiences of mental health recovery: reflections and recommendations on using digital storytelling	Mcdonough, S./ 2019 (17)	B	C	A	B	C	C
A pediatric digital storytelling system for third year medical students: the virtual pediatric patients	D'Alessandro, D.M./ 2004 (45)	B	C	A	A	A	C
Digital storytelling for reflection in undergraduate medical education: a pilot study	Sandars, J. / 2009 (15)	C	C	A	B	A	C
Development and evaluation of a digistory^3^ about autistic spectrum disorder - a pilot study	Codd, A./ 2018 (46)	C	C	A	A	B	C
Digital storytelling: an innovative technological approach to nursing education	Price, D./ 2015 (5)	B	C	A	C	A	C
Creation and Online Use of Patient-Centered Videos, Digital Storytelling, and Interactive Self-testing Questions for Teaching Pathophysiology	DeLenardo, S./ 2019 (47)	C	C	A	A	A	C
Challenging the shock of reality through digital storytelling	Stacey, G./ 2011 (14)	B	C	A	B	A	C
The use of digital storytelling in nursing education, case of turkey: Web 2.0 practice	Tatli, Z./ 2017 (23)	C	C	A	C	C	C
Digital stories: Incorporating narrative pedagogy	Gazarian, P.K./ 2010 (20)	B	C	A	A	B	C
Using a digital storytelling assignment to teach public health advocacy	de Castro, A.B. / 2017 (48)	B	C	A	A	A	C
A family nursing educational intervention supports nurses and families in an adult intensive care unit	Eggenberger, S.K./ 2016 (25)	C	B	A	B	A	C
Learning from clinical placement experience: Analyzing nursing students’ final reflections in a digital storytelling activity	Paliadelis, P./ 2016 (49)	C	C	A	B	B	C
Development of a digital storytelling resource to support children's nursing students in neonatal care	Petty, J./ 2017 (24)	B	C	A	B	A	C
Dangling conversations: Reflections on the process of creating digital stories during a workshop with people with early-stage dementia	Stenhouse, R./ 2012 (50)	B	C	A	B	B	C
Digital storytelling in clinical replacement studies: Nursing students' experiences	Urstad, K.H./ 2018 (16)	C	C	A	B	A	C
Omnipresent learning via interactive media	Lyons, T. / 2013 (18)	C	C	A	B	A	C
Feasibility and Acceptability of a 3-Day Group-Based Digital Storytelling Workshop among Caregivers of Allogeneic Hematopoietic Cell Transplantation Patients: A Mixed-Methods Approach	Kim, W./ 2019 (51)	C	C	A	B	B	C
Moms Supporting Moms: Digital Storytelling with Peer Mentors in Recovery from Substance Use	Paterno, M.T./ 2018 (52)	B	C	A	B	B	C
Web-based survey on the effect of digital storytelling on empowering women to seek help for urogenital atrophy	Cumming, G.P./ 2010 (53)	B	C	A	A	A	C
Simulating social work practice online with digital storytelling: challenges and opportunities	Goldingay, S./ 2018 (54)	C	C	A	B	A	C
An interdisciplinary approach to the development of professional identity through digital storytelling in health and social care and teacher education	Marín, V.I./ 2018 (19)	B	C	B	B	A	C

1 Community-based participatory research;

2 Human Immunodeficiency Virus/ Acquired Immunodeficiency Syndrome;

3 Digital story

QUESTS is a multidimensional grading approach in which articles are assessed in six dimensions. In contrary to the unidimensional grading scheme which is used in evidence-based medicine (EBM), it scores the studies in accordance with expert opinions, descriptive studies, quasi experimental studies, controlled studies without randomization, randomized control trials, and meta-analysis. The QUESTS’ six dimensions are clarified in Table 1 ( 30 ). 

**Ethics approval:** This study approved ethically by Imam Khomeini hospital complex, Tehran University of Medical Sciences with ID. IR.TUMS.IKHC.REC.1398.238. 

## Results

**Figure 1:**
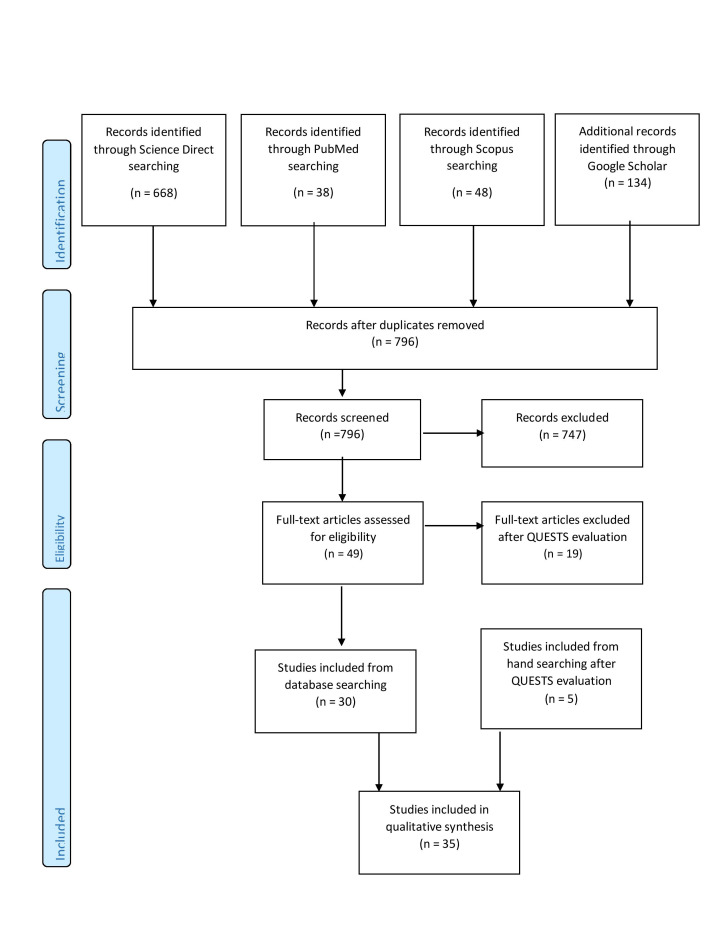
PRISMA flowchart for including papers in the review

The number of studies retrieved after screening and evaluation was 35 (Figure 1). The QUESTS continuum for each study varied from low (A grade), medium (B grade) to high (C grade). The studies were included in the review if they obtained at least three scores of B or C in any dimensions of QUESTS. In this perspective, all 35 studies which were retrieved through screening were thus included in the review. 

In this section, first of all the studies were categorized by field of health profession. The selected articles were in six fields of health profession. Twelve studies in Nursing Education, three in Medical Education, two in Community Health Education, two in Social Work Education, 13 in Health Promotion, and three in Patient Education.

The detailed bibliographic information of articles and classification by aim, process and performers of DST are summarized in Table 3.

**Table 3:** Bibliographic Information and Classification of Retrieved Articles by Application, Process, and Producer

Field of study	First author/ Year of publication	Aim of study	Study design	Implication of DST	Producers of DST	Process of DST	Country
Community Health Education	Cueva, M./2013 (31)	To learn how CHWs^1^ in Alaska perceive digital storytelling as a component of a cancer education curriculum and as a culturally respectful tool for sharing cancer-related health messages	Developmental design	To share cancer-related health messages as a tool in cancer education curriculum	Community health workers	Participants synthesize and integrate their cancer understandings with a personal narrative to create their own culturally relevant cancer health message. Not mention anything else about the process of DST^2^.	**USA**
	Holliday, C.E./ 2016 (32)	To clarify the needs of tribal community members by DST to prevent suicide and substance use	Evaluation design	As a tool to data collection in community assessment	Youth and other community members	Visually documenting things, people, places, and items, sharing points of view and discussing, digitalizing and sharing them.	**USA**
Health Promotion	Rimando, M./ 2015 (33)	To promote health behavior change, community health education program and a health advocacy campaign for a specific target population	Developmental design	To empower client involved in counseling process	Students	Writing scenario and video, getting feedback, finalizing videos, grading videos by the instructor by a rubric, presenting videos and evaluating and providing the main message learned.	**USA**
	Briant, K.J./ 2016 (34)	To investigate if DST is a good tool for Hispanics/ Latinos to share their experiences with diseases	Developmental design	As a tool to share experiences, as a healing channel to reflect on experience and find support in community	Community members	Sharing ideas, receiving feedback, writing script, revising, making storyboard, recording voice, digitalizing the story and sharing digital stories.	**USA**
	Cueva, M./ 2015 (35)	To investigating community members’ perspectives about digital storytelling	Qualitative design	As an educational tool to change the health behavior	CHA/P^3^ course participants (community members that participate and train for health promotion)	Writing a 250-300 words script, recording narratives, adding photos and music, digitalizing with help of two course facilitators. The final products are two- to three-minute digital story.	**USA**
	DiFulvio, G.T. / 2016 (36)	To investigate the outcomes associated with the DST process	Evaluation study	As a tool to change attitude and values, self-esteem, social support and empowerment	Puerto rican latinas between the ages of 15 to 21	This article referenced another article from one of their authors to describe the DST process. This process was: writing the script, sharing and listening to others. revising the script, recording voice over, storyboarding and digitalizing that. Finally showing the digital story happened and viewers offer some recommendation (44)	**New England, USA**
	Fiddian-Green, A./ 2017 (37)	1. To illustrate the value of DST for sexual health intervention design 2. To inform the development of meaningful health promotion efforts for Puerto Rican Latina youth	Case study	As a part of a culture centered approach and as a tool to change the sexual values and practice	Puerto rican latinas between the ages of 15 to 21	As DiFulvio, G.T. study (36)	**USA**
	Gilliam, M./ 2012 (38)	To overview of the institute program in digital media and sexuality	Case report	As a tool to improve sexual and emotional change	American youth	Producing the script, integrating images, applying effects and producing a digital story through story circle.	**USA**
	Njeru, J.W./ 2015 (39)	To develop a diabetes digital storytelling intervention for immigrants and refugees	Case report	As an educational intervention for immigrants and refugees (in context of chronic disease and user with limited language deficiency)	Researcher team	Conducting some surveys and focus groups among community members with type II diabetes, recording session when each member told his/her experience, subscribing and using for other community members.	**USA**
	Weigensberg, M.J./ 2014 (40)	1) To determine the effects of the mind-body modality of Interactive Guided Imagery on insulin resistance, eating and physical activity behaviors, stress and stress biomarkers; 2) To explore the role of intervention-related changes in stress and stress biomarkers on changes in metabolic outcomes	Randomized control trial	As a tool to promote lifestyle behavior change	Latino adolescents	Not mention	**USA**
	Wexler, L./ 2013 (41)	To use DST as a health promotion strategy	Case study	As a platform to reflect on and represent the lives, important relationships and achievements / As a health promotion strategy	Alaska natives	Writing a script, recording a voiceover narration, digitalization and showing the final digital story.	**USA**
	Wieland, M.L./ 2017 (41)	To examine the effectiveness of a digital storytelling for immigrants and refugees with type 2 diabetes mellitus	Quasi- Experimental design	To promote confidence and motivation of immigrants and refugees in diabetes mellitus management	Participants overcoming obstacles to health promoting behaviors	Referenced to Njeru, J.W. article (41).	**USA**
	Ezegbe, B./ 2018 (43)	To determine the efficacy of a digital storytelling on knowledge and perception of risk of HIV/AIDS^4^ among schoolchildren	Randomized control trial	As a tool to promote knowledge and perception of risk of HIV/AIDS among schoolchildren	Not mentioned. (But not participants)	Recording the people’s lived experiences related to HIV/AIDS as a video before the study, watching this video individually/ or in group meeting by participants.	**Nigeria**
	Gubrium, A.C./ 2009 (44)	To determine the effectiveness of a program to improve the health and quality of life of low-income African-American men	Case report	As a tool to improve the health and quality of life of low-income African-American men	African-American men	Scripting, reading and sharing the script through story circle (discuss and feedback), selecting photo and video clips, recording a narration, adding a title/credits/transition/and effects, screening digital stories and discussing about that.	**USA**
	Mcdonough, S./ 2019 (17)	To describe the process of project and the results of follow up Three years conducted after original work.	Survey	As a tool to share experience	Immigrants & refugees with lived experience of mental ill health and recovery	Sharing the idea in story circle, scripting, making photo story, creating images, making three to four minutes narration, searching and creating sound track, making a film by professional film makers, showing films to participants and reflecting.	**UK, Australia**
Medical Education	D'Alessandro D.M./ 2004 (45)	To describe the development of a CBPS^5^	Developmental design	As a CBPS	Student, professors, science educator and professional storyteller	Writing the first-person patient scenario by a second-year medical student, conducting a literature review and writing a patient's actual disease process by practicing pediatrician, reviewing the preliminary story by a science educator and professional storyteller, digitalizing all media by a medical student.	**USA**
	Sandars, J. / 2009 (15)	To investigate the potential effects of digital storytelling to engage students in reflection	Qualitative exploratory design	To engage students in reflection	Medical students	Capturing the images by cellphone, creation Digital stories using PowerPoint software, presenting individual digital stories and reflecting.	**UK**
	Codd, A./ 2018 (46)	To develop, implement and evaluate the digistories^6^ for supporting patient centered learning	Pre-post design	To encourage confidence to engage in future clinical encounter and support patient centered learning	A student researcher	Recording the narrative of sam, a mother of child with ASD^7^, editing and piloting the recording on medical student to ensure retained key points, digitalizing as a cartoon, piloting and editing again, presenting to students as a package.	**USA**
Nursing Education	Price, D./ 2015 (5)	1. To investigate of how digital storytelling effects learning processes 2. To investigate of how educators can effectively scaffold learners’ creation of digital stories to facilitate peer learning and sharing	Pre-experimental	To promote deeper understanding in nursing students about palliative care concepts	Nursing students	Students using VoiceThread, an online software, uploaded video, document, PPT^8^ and …, recorded voice and produced a five-minute first-person digital story that shared and discussed within Small group (but not live). The coaching, grading, and ranking, all of them are done through software.	**USA**
	DeLenardo, S./ 2019 (47)	To restructure and personalize the study of human disease by scripting and producing videos	One-group Pre-post design	As a tool to engage students in self-directed, online learning and to restructure and personalize the study of human disease	Not exactly mentioned. The role of students was only actor/actress.	Writing scripts, storyboarding, recording students acting, video and audio recording and finally producing five- to nine-minute films.	**Canada**
	Stacey, G./ 2011 (14)	To report the educational development to eliminate negative experiences of students’ transition to workplace	Developmental design	To eliminate negative experiences of students’ transition to work place	Newly qualified nurses	Considering the elements of a good story; sharing new ideas through story circle, editing, refining and recording the story; choosing photos and creating movies.	**UK**
	Tatli, Z./ 2017 (23)	To evaluate the effects of digital storytelling and hand-drawn storytelling boards on nurses’ empathy and case analyzing	Mixed method	To effect on nurses’ empathy and case analyzing	Nursing students	Writing script, storyboarding, locating multimedia, structuring digital story, sharing and analyzing the process by survey and rubric.	**Turkey**
	Gazarian, P.K./ 2010 (20)	To describe the use of DST for students’ clinical thinking enhancement	Developmental design	To enhance students’ clinical thinking	Nursing students	Writing script, adding multimedia, presenting digital stories (in three to five minutes), discussing and getting feedback.	**USA**
	de Castro, A.B. / 2017 (48)	To describe how digital story making can be utilized as an academic assignment to teach public health advocacy	Case report	As an academic assignment to teach public health advocacy	Nursing students with help digital media staff	Selecting an issue, storyboarding, sharing the initial storyboard and discussing, digitalizing story. Received a scoring the final digital story.	**USA**
	Eggenberger, S.K./ 2016 (25)	To examine the influence of DST on nurses’ attitudes towards and confidence in providing family care	Mixed method	As a tool to promote nurses’ attitudes towards and confidence in providing family care	Research team	Not mentioned	**Australia**
	Paliadelis, P./ 2016 (49)	To explore whether reflecting on previous clinical events support students’ transition into practice	Qualitative method: (content analysis)	As a tool for reflecting and supporting students’ transition into practice	students	uploading students’ narratives on online forum, analyzing that narrative by researchers.	**Australia**
	Petty, J./ 2017 (24)	To utilize student experiences by DST to teach other learners	Evaluation design	As a tool to teach other learners about other students’ experience (To share experience)	Co-authors	Interviewed and recorded students’ experiences, storyboarding and rewriting, creating multimedia with recording subjects, sending recorded stories to students to verify and comments, publishing online.	**England**
	Stenhouse, R./ 2012 (50)	To develop a learning package to help nurses who work with people with dementia	Case report	As a tool to collect patient experiences and help nurses to engage with those experinces	People with early-stage dementia	Writing script, scanning all images, reading scripts to the groupmates, recording voice, selecting images or videos, digitalizing, choosing music and showing the stories.	**Scotland**
	Urstad, K.H./ 2018 (16)	To investigate the nursing students' experiences with digital storytelling creation	Explorative qualitative design	As a tool for reflection during clinical experience	Nursing students	Identifying a story, sharing an initial story, film production in the technical session, individual manuscript production and digitalization, group presentation and discussion.	**Norway**
	Lyons, T. / 2013 (18)	To facilitating discussion board activities and digital storytelling and evaluating the participation in these active learning strategies	Mixed method	As an active learning strategy	Students, librarian, and instructor	In an interactive online threat, making a story about own experience about research issues.	**USA**
Patient education	Kim, W./ 2019 (51)	To assess the feasibility and acceptability of DST workshop	Mixed method	As a tool to decrease anxiety and depression	Patients	Sharing the oral story, writing script, storyboarding, recording narration, taking/ making photos or videos, combining all materials and producing the final product. Sharing and discussing the digital story were the final steps.	**USA**
	Paterno, M.T./ 2018 (52)	To assess the feasibility of DST for improvement in women with history of perinatal SUD^9^	Case study	As a mechanism to understand the substance use disease and recover from that	Women with lived experience of perinatal SUD	Not mentioned exactly. Three authors of this study previously participated in workshop hold by CDS^10^.	**USA**
	Cumming, G.P./ 2010 (53)	To evaluate an online digital story to empower women suffering from urogenital atrophy to seek health professional advice	survey	As a tool to empower women suffering from urogenital atrophy to seek health professional advice	Internet design shop	The clip ‘Breaking the Silence’ that was five minutes in length (10 Mb^11^) in WMV^12^ format used for observation.	**UK**
Social Work Education	Goldingay, S./ 2018 (54)	To prepare students for challenging of modern social work practice	Mixed methods evaluative study	To prepare students for the challenges of modern social work practice	The social work team of university & learning designers & digital resource producers	The 14-minute video-diary with staring Evelyn talking directly to the camera (to the students) in several different situations around her home. These products were used for students' instruction.	**Australia**
	Marín, V.I./ 2018 (19)	To investigate the use of digital tools on learning and promoting reflection on professional roles	Multiple case study	To promote reflection on professional roles and learning of students and identity construction	Undergraduate health and Social Care students & undergraduate primary education teacher & postgraduate secondary education teacher	Building storytelling artifacts, showing the artifacts in the class, getting feedback, revising artifacts, reflecting on identity, giving feedback on each group.	**Spain, UK**

1 Autism spectrum disorder;

2 Power-Point presentation;

3 Substance use disease;

4 Center for Digital Storytelling;

5 Mega byte;

6 Windows Media Video;

7 Community health worker;

8 Digital Storytelling;

9 Community Health Aide/Practitioner;

10 Human Immunodeficiency Virus/ Acquired Immunodeficiency Syndrome;

11 Computer-based patient simulations;

12 Digital story

### Community Health Education

Among 35 studies, there were two studies which were conducted in this field. The participants in the DST were community health workers ( 31 ) and community members ( 32 ). Cueva (2013) used DST as a tool for sharing health-related experiences in cancer curriculum. The process of DST is not mentioned in this study ( 31 ). Holliday (2016) used DST for community assessment, so the DST process was recommended for the documentation of items as well as sharing and digitalizing them ( 32 ). 

### Health Promotion

Thirteen included studies were conducted on health promotion. In the Rimando’s study ( 33 ), he process of digital storytelling refers to students writing scripts, making videos, getting feedback, scoring videos, and presenting and showing the important message of that story. Their goal was to empower the clients involved in counselling process. 

Briant ( 34 ) has discussed the process of sharing ideas, getting feedback, writing scripts, producing storyboards, recording videos, and digitizing and sharing them for reflection.

The process of digital storytelling in Cueva’s study ( 35 ) was to write 250- to 300-word scripts, record narration, add photos, and turn them into digital stories with the help of facilitators. This was done by community members mainly focused on their behavior change.

DiFulvio ( 36 ) and Fiddian-Green ( 37 ) conducted DST through scripting, subscription and feedback, editing, narration, storyboard production, digital storytelling, and finally sharing and gaining suggestions from others. Performers of DST in these studies were Puerto Rican Latinas between the ages of 15 to 21. They produced digital stories to change attitude and values, self-esteem, social support and empowerment ( 44 ), and to change the sexual values and practice ( 37 ). Furthermore, Gubrium ( 44 ) and Mcdonough ( 17 ) used DST to improve the health and quality of life ( 44 ) and share the experiences ( 17 ). Gubrium did not mention storyboarding ( 44 ). 

In some studies, community members produced a digital story in steps including producing the script, recording a narration, and digitalizing it. They used DST as a tool to improve sexual and emotional behavior ( 38 ), sharing experiences, reflecting, and health promotion strategy ( 41 ). 

In Njeru ( 39 ) and Wieland’s studies ( 42 ), the research team produced a digital story in which they recorded experiences of people with type II diabetes to educate immigrants and refugees with language insufficiency ( 39 ), and to promote confidence and motivation in them ( 42 ). Like Njeru, Ezegbe ( 43 ) also referred to producing films to promote knowledge and perception of the risk of HIV/AIDS among school children. 

### Medical Education

Three studies were in this field. D'Alessandro ( 45 ) referred to DST as a patient simulation in which a medical student, their professors, and a professional storyteller collaborated. The patient story script was prepared by the medical student and then, based on a literature search, the disease process was explained under the supervision of the professor and the storyteller, and finally the story was compiled digitally by the student.

In another study, Sandars ( 15 ) used DST as a tool to engage medical students in reflection. In this study, medical students took photos and then produced a digital story using PowerPoint software. After that, they presented it and reflected on it. In Codd's study ( 46 ), a member of the research team was tasked to produce a digital story. He recorded the words of a child’s mother with ASD and edited it into a cartoon. This cartoon was given to other students as an educational package so that they could gain the required confidence and preparation for future encounters.

### Nursing Education

In nursing education, 12 articles have been published in DST. Price ( 5 ) used DST to enhance nursing students' deep understanding of the concepts of palliative care. They uploaded videos to an online software, produced a five-minute digital story, and discussed the digital story shared offline. 

DeLenardo ( 47 ) in her study applied DST as a tool for student participation in self-directed online learning. In this study, the students only played the role of story actors. The process of digital storytelling was accomplished in the form of writing a script, producing a storyboard, recording audio and video, role playing of students, and finally producing a five to nine-minute story.

In Stacey’s study ( 14 ), new-qualified nurses presented their experiences from their transition from the student stage to the real work stage through digital storytelling. In the story cycle, they shared their experiences, edited and recorded them, and finally turned them into films. Like Stacy, Paliadelis ( 49 ) also supported the students transition to work setting as well as their reflection by previously produced podcasts. Urstad ( 55 ) applied DST for students’ reflection as well. In her study, the nursing students had to choose a story and turn it into a movie. Then, they shared and discussed it. Furthermore, based on interviews with nursing students, Petty ( 24 ) recorded their experience, made a storyboard, and then released a film online to educate other students. 

In her study, Tatli ( 23 ) used digital storytelling to compare storyboarding manually to compare empathy and case analysis skills in two groups. The students in the DST group wrote a script, then compiled a storyboard, made and shared the digital story, and evaluated it using rubrics. 

Another study discussed the application of DST to promote critical thinking. The process of digital storytelling in this study was writing a script, adding multimedia, producing a three- to five- minute digital story, discussing it, and giving feedback on it ( 55 , 56 ). DST also was used as an academic assignment to teach public health advocacy to nursing students who chose a topic and created a storyboard, shared, and discussed it. Finally, with the help of media staff, they produced a digital story and received a score ( 48 ). 

Eggenberger, et al. ( 25 ) produced a digital story in order to promote students' attitudes toward family care and increase their self-confidence. Also, in nursing education, patients with dementia turned their personal experiences into digital story through script writing, photo selection, group script sharing, audio and video recording, and digitization to be used later in student education.

Finally, in another study, nursing students, along with their professor and a librarian, produced the digital stories of their experiences with research issues through an online net. In this study, storytelling was mentioned as an active learning strategy ( 18 ).

### Patient Education

Kim ( 51 ), Paterno ( 52 ), and Cumming ( 53 ) conducted some researches in this field. Kim, Paterno, and Cumming used DST as a tool to reduce patients' anxiety and depression, to better understanding of pregnant women about substance abuse in pregnancy and their recovery, and to empower women and help them seek professional health advice, respectively. In Kim and Paterno’s studies, the patients produced a digital story themselves ( 51 , 52 ). On the other hand, Cumming ( 53 ) used a five-minute produced film to educate patients. 

### Social Work Education

Of the two studies conducted in this field, one was aimed at preparing students for the challenges of social work ( 54 ) and the other was to promote reflection on professional roles and the construction of students' identities ( 19 ). In Goldingay’s study ( 54 ), the research team provided a 14-minute film in which a person was talking to the camera in various situations around her house. On the contrary, Marin ( 19 ) used a method in which students, along with others, generated storytelling artifacts, displayed them in the class, received feedback, and reflected on their identity. 

## Discussion

This systematized review was conducted to answer this question “how DST is used in health profession education”. In this regard, the applications and process of DST in health profession education, the digital story producer and the health profession field in which DST was used were identified. From the vantage of the DST producers in the retrieved studies, there is a wide range of health education’s stakeholders including public ( 34 ), students ( 23 ), teachers ( 45 ) and patients ( 50 ), who created any short movies with or without narration. The creators of digital stories in some studies were the learning target population themselves; however, in some similar studies, digital stories were produced by someone else to teach the target population. In some studies, the process of DST was an integral part of learning ( 5 , 14 , 15 , 23 , 32 - 34 , 38 , 44 , 45 , 47 ), while there was no emphasis on the DST process in some other studies ( 18 , 19 , 24 , 31 , 35 , 47 , 52 - 55 ). There are various fields in health profession in which DST is applied. 

According to the results of this study, there was flexibility in DST usage which could be due to multimodality of this educational technology. Its multimodal nature makes this method suitable for different learning styles ( 10 , 23 ).

Furthermore, in some studies, the producers of DS were different from the groups that researchers aimed to investigate the effects of DST on. In other words, the producers and the users are not the same. For example, Stacey ( 14 ) indicated that newly qualified nurses presented their experience in DST by showing the stories to other students so that they could overcome the reality shock. Likewise, D'Alessandro ( 45 ) explained a DST system that applied digital stories, having been already produced by others. In addition, Stenhouse ( 50 ) mentioned the digital stories produced by people with dementia which later were used in students’ education. All these digital stories were used in academic education for students, while the students themselves were not the producers. According to CDS ( 9 ), DST, however, is a first person experience. Therefore, these studies are not in accordance with original definition of DST. 

Investigating the DST process, which was the other purpose of this review, is in line with the previous findings. Some studies utilized a short movie/story produced before or at the same time of studying to use for education in another target population ( 14 , 24 , 25 , 35 , 39 , 42 , 43 , 45 - 47 , 50 , 53 , 54 ). While Fenton ( 57 ) employed the “Digital Learning Object” (DLO) term for such usage, these studies have used DST term. 

The first model of DST, which is pioneered by Lambert in CDS, emphasized the process of DST already introduced in the introduction. He focused on the DST in a story circle in which individuals work with each other, tell and read their stories, react, and get reaction of others and edit the story structure ( 9 ). Inspired by Lambert, Robin ( 1 ) introduced a process for DST to use in educational contexts. Reflecting on the process of DST, it is probably an accurate conclusion that the process of DST is as important as or even more than its product since every student can produce a simple movie by any accessible application/ software regardless of the DST process or its elements. 

This means when DST is applied in a study, it is expected that the DST process should be taken into consideration. In spite of this, in some studies, digital storytelling has been referred to as a story/film presentation to the target population produced by someone else ( 14 , 24 , 25 , 35 , 39 , 42 , 43 , 45 - 47 , 50 , 53 , 54 ). In other words, it was used as a research-based/teacher-based approach. For example, Mcdonough ( 17 ) in her study described a project report that the refugees provided a script while filmmakers digitalized it. In this regard, the refugees as the members of DST workshop only produced a script not a digital story. On the other hand, Sandars ( 15 ) in a qualitative study describes experiences of DST which are all performed by students. Perhaps these two different approaches in DST are related to the type of participants (refugees - medical students) and the goals (sharing experience- reflection) of these studies.

In the case of the health profession field in which DST is used, the retrieved studies showed that while it has a wide application in many fields, there are limited studies conducted on higher education in health sciences. 

Given what is already mentioned, it seems DST has the potential to be used by various people and in many fields. Robin ( 10 ) contends that DST is compatible with various learning styles. Also, there are studies that reported the results of DST application in language education ( 58 , 59 ), art education ( 40, 60 ) and information systems learning ( 61 ). Sawyer ( 62 ) introduced DST to influence children and high school students. Besides, utilizing DST in higher education is reported ( 63 ) in literature.

In addition, the studies reviewed all foster deep understanding ( 5 ), reflection ( 15 , 19 ), empathy, case analysis ( 23 ), critical thinking ( 20 ), identity construction ( 19 ), creativity, innovation, involvement in learning ( 33 ), empowerment in requesting social support, increasing self-esteem, attitude change ( 64 ), improvement in clinical thinking ( 20 ), and clinical skills ( 45 ). Definitely, educators must know that their students will become more successful in case of more engagement, sense of belonging and increase in positive behaviors, and achievement occurrence ( 65 ). Moreover, education should improve critical and probably more creative thinking ( 66 ). It seems these consequences occur when the person does the DST her/himself. 

Nevertheless, some studies utilized DST in different designs. For instance, Price ( 5 ) refers to the process of uploading documents, PPT, video, sound recording, sharing, and commenting through VoiceThrea. The discussion took place asynchronously; meanwhile, the feedback discussion from participants could be written or oral although the steps such as scripting and storyboarding of DST have not been clearly stated. Stacey ( 14 ) explained DST as a reflection tool for new qualified nurses to share their experiences by DST. The steps of DST in this study have been defined as creating, editing and recording a story, selecting photos, and thus creating a film. A summary of other methods is given in Table 3. While different DST stages considered in various studies, it seems almost all of them had focused on story circle and feedback, reflection, reviewing, editing and re-editing, which is important in DST. 

In case of the implications of DST, there were many different methods shown in Table 3. These implications differ from constructing knowledge ( 47 ), sharing knowledge ( 17 , 24 , 31 , 34 ), changing attitude ( 25 , 64 ), values ( 64 ), and behavior ( 35 , 38 , 40 ) and promoting the substantial skills such as reflection ( 15 , 19 , 49 ), critical thinking ( 20 ), empathy ( 23 ), motivation, and confidence ( 42 ). Moreover, effects on some psychological factors such as depression and anxiety ( 51 ) are considered. Of these studies, Tatli ( 23 ) investigated the effects of DST on empathy in nursing students even though the effects of making storyboard by computer and hand was investigated in two groups and on the empathy. It could be concluded that Tatli has more intended to study the DST process. 

Albeit most of these implications are not used in academic education, the results indicate that DST is a powerful educational approach that has potentials to be applied at various learning levels including higher order of cognitive, affective and psychomotor as well as various levels of education in different subjects in many disciplines in health profession. 

There are three limitations for this study. The first is the lack of quality assessment of the included manuscripts by two reviewers. The second is related to a non-comprehensive search. In addition, among the included studies, there were a few experimental studies with a control group ( 40 , 42 , 43 ), so there is some uncertainty on the results of other studies which can affect the quality of the synthesized results. 

## Conclusions

This systematized review investigated 35 articles which employed DST as an educational method and technology. The results indicated that DST had some applications in different subjects in different fields of health professions. According to the definition of CDS, digital storytelling involves writing a script to producing a digital story by either one individual or a group. So, there is a difference between DST and digital story and as the present review showed in some studies the digital story was produced by one person or group and used for educational purposes in another group. Conversely, in some studies, the learners themselves did the process of DST. Therefore, researchers should consider the correct use of this term in their studies. Although few experimental and high-quality studies have been conducted in this area, further both quantitative and qualitative research are suggested to evaluate the effectiveness of this technology on different variables influential to learning and the best efficient way to use DST.
